# Disease-Modifying Symptomatic Treatment (DMST) Potential of Cannabinoids in Patients with Multiple Sclerosis

**DOI:** 10.2174/011570159X329058240820070701

**Published:** 2024-09-13

**Authors:** Antonio Bruno, Pietro Annovazzi, Marinella Clerico, Eleonora Cocco, Antonella Conte, Girolama Alessandra Marfia, Marco Salvetti, Valentina Tomassini, Valentina Torri Clerici, Rocco Totaro, Ettore Dolcetti, Diego Centonze

**Affiliations:** 1 Neurology Unit, IRCCS Neuromed, Pozzilli (IS), Italy;; 2 Neuroimmunology Unit, Multiple Sclerosis Centre ASST Valle Olona, Gallarate Hospital, Gallarate (VA), Italy;; 3 Clinical and Biological Sciences Department, University of Torino, Cagliari, Italy;; 4 Centro Sclerosi Multipla, Department of Medical Science and Public Health, University of Cagliari, Cagliari, Italy;; 5 Department of Human Neurosciences, Sapienza, University of Rome, Rome, Italy;; 6 Department of Systems Medicine, Tor Vergata University, Rome, Italy;; 7 Multiple Sclerosis Clinical and Research Unit, Tor Vergata University Hospital, Rome, Italy;; 8 Centre for Experimental Neurological Therapies (CENTERS), Department of Neurosciences, Mental Health and Sensory Organs, Sapienza University of Rome, Rome, Italy;; 9 Institute of Advanced Biomedical Technologies (ITAB), Department of Neurosciences, Imaging and Clinical Sciences, University G. d’Annunzio of Chieti-Pescara, Chieti, Abruzzo, Italy;; 10 Neuroimmunology Unit, IRCCS Istituto Neurologico C. Besta, Milan, Italy;; 11 Demyelinating Disease Center, Department of Neurology, San Salvatore Hospital, L’Aquila, Italy

**Keywords:** Cannabinoid, spasticity plus syndrome, multiple sclerosis, disease modifying therapies, neuroprotective effects, symptomatic therapies

## Abstract

With the recent introduction of a number of highly effective disease-modifying treatments (DMTs) and the resulting almost complete prevention of acute relapses in many patients with multiple sclerosis (MS), the interest of MS clinicians has gradually shifted from relapse prevention to counteraction of disease progression and the treatment of residual symptoms. Targeting the cannabinoid system with nabiximols is an approved and effective strategy for the treatment of spasticity secondary to MS. Recently, the concept of spasticity plus syndrome (SPS) was introduced to account for the evidence that spasticity often appears in MS patients in clusters with other symptoms (such as pain, bladder dysfunction, sleep, and mood disorders), where cannabinoids can also be effective due to their broader action on many immune and neuronal functions. Interestingly, outside these symptomatic benefits, extensive pre-clinical and clinical research indicated how the modulation of the cannabinoid system results in significant anti-inflammatory and neuroprotective effects, all potentially relevant for MS disease control. This evidence makes nabiximols a potential disease modifying symptomatic treatment (DMST), a concept introduced in an attempt to overcome the often artificial distinction between DMTs and symptomatic therapies (STs).

## 1 INTRODUCTION

### 1.1 The Concept of Disease-modifying Symptomatic Treatment (DMST) in Multiple Sclerosis

The recent introduction of highly effective disease-modifying treatments (DMTs) in the clinical practice of relapsing-remitting multiple sclerosis (RRMS) has drastically reduced the frequency of acute inflammatory episodes and relapses [1], gradually shifting the interest of clinicians toward preventing disease progression and treating associated symptoms [2, 3]. Interestingly, highly effective DMTs often result not only in relapse prevention but also in the amelioration of pre-existing symptoms, such as fatigue, cognition, and motor disability, leading in some cases to the unexpected reduction of their Expanded Disability Status Scale (EDSS) score [4, 5]. On the other hand, many pharmacological and non-pharmacological symptomatic treatments (ST) also exert anti-inflammatory and neuroprotective effects, as in the case of antidepressant drugs, physical rehabilitation, and cannabinoids. Thus suggesting that the distinction between DMTs and STs is somehow artificial. To account for the symptomatic effects of classical DMTs and for the disease-modifying potential of approved STs, a group of MS specialists gathered in the DMSTs in MS Italian Study Group, with the specific aim of overcoming, where possible, the classical distinction between DMTs and STs to optimize MS treatment. The Group regularly met in Rome in 2022 and 2023 to discuss the DMST potential of specific pharmacological and non-pharmacological interventions. This article summarizes the output of a meeting held on April 21^st^ and 22^nd^, 2023, focusing on the pharmacological treatment of patients with MS (pwMS) with cannabinoids.

## 2 GENERAL CONSIDERATIONS

The new paradigm of spasticity plus syndrome (SPS) proposes that spasticity and other accompanying symptoms (*e.g*., spasms/cramps, pain, bladder dysfunction, sleep disturbances, and fatigue) constitute a cluster of clinical manifestations independently linked by a common underlying pathophysiology [6-8]. As a practical consequence, a single drug could act on the entire cluster of symptoms that constitutes SPS [9]. In the early 2000s, two randomized controlled trials showed that two compounds extracted from the Cannabis sativa, delta-9-tetrahydrocannabinol (THC) and cannabidiol (CBD), improve gait control, balance, spasm frequency, and insomnia [10, 11]. Nabiximols (Sativex^®^), an oral mucosal spray that comprises a balanced 1:1 ratio mixture of THC and CBD, is approved for the treatment of moderate to severe spasticity in pwMS [3]. 40% of initial responders to nabiximols achieve meaningful and durable symptomatic improvement of spasticity, lasting for months or years of continued treatment [7] as estimated by several studies [11-14], also in patients with progressive MS (PMS) with severe spasticity at baseline [15]. Long-term treatment with nabiximols also showed efficacy in treating other symptoms belonging to SPS, including sleep disturbances [8], muscle stiffness [16], spasms/cramps [8], and neuropathic pain [17-19] modulating pain threshold in pwMS [20]. Current literature also demonstrated that nabiximols are effective in the control of bladder symptom severity nocturia [21, 22] and enhance urodynamic parameters independently from spasticity improvement [3], consistently with the SPS theory [6]. In addition, compelling experimental data demonstrated that cannabinoids modulate the overreactive immune system and synaptic dysfunction on the basis of the pathophysiology of MS and of its murine model, the experimental autoimmune encephalomyelitis (EAE) [23]. In this article, we summarize the role of cannabinoids in the pathophysiology of MS and discuss further perspectives and potential clinical uses of nabiximols and cannabinoids as DMSTs.

## 3 CANNABINOIDS AND THE PATHOPHYSIOLOGY OF MS

The dysregulation of the endocannabinoid system (ECS) in MS and EAE has been extensively documented by clinical and preclinical literature [24]. Increased levels of anandamide (arachidonoyl-ethanol-amide or AEA) have been documented in both relapsing pwMS and EAE mice [25]. Moreover, cannabinoid-receptor-1 (CB1R) genetic ablation exacerbates the neurodegenerative damage of EAE further, suggesting a functional role for cannabinoids receptors in MS pathophysiology [26]. Since the 2000s, preclinical studies demonstrated that cannabinoids counteract the neurodegenerative process that leads to chronic disability in EAE [27], reducing excitotoxicity and oxidative stress and promoting neurogenesis [28]. Moreover, cannabinoids inhibit the breakdown of myelin, preventing or reversing the demyelination process [24]. Several studies have shown that *in vivo* treatment with cannabinoids modulates key immune mechanisms in EAE mice [24]. AEA significantly attenuates neuroinflammation [25], inhibiting microglial activation and reducing the release of interleukin (IL)-23 and IL-12 [29], as well as of IL-1β and IL-6 [30] released by myeloid dendritic cells [24]. 2-Arachidonoylglycerol (2-AG), another endocannabinoid, delays the onset of acute and chronic EAE, inducing the recruitment of anti-inflammatory macrophages [31]. Interestingly, CBD lowers inflammation, microglia activation, and T-lymphocyte recruitment in the spinal cord [32], enhancing interferon (IFN)-γ-dependent anti-proliferative responses, suppressing proinflammatory Th17 responses and preventing antigen presentation [32]. In 2015, two studies were conducted on EAE mice treated with a nabiximols-like combination of phytocannabinoids, showing the promotion of myelin repair and reducing cell infiltrates in the spinal cord [33], microglial activation, and IL-1β gene expression [34].

## 4 CANNABINOIDS AND THE MODULATION OF INFLAMMATORY SYNAPTOPATHY IN MS

**Fig. (1):**
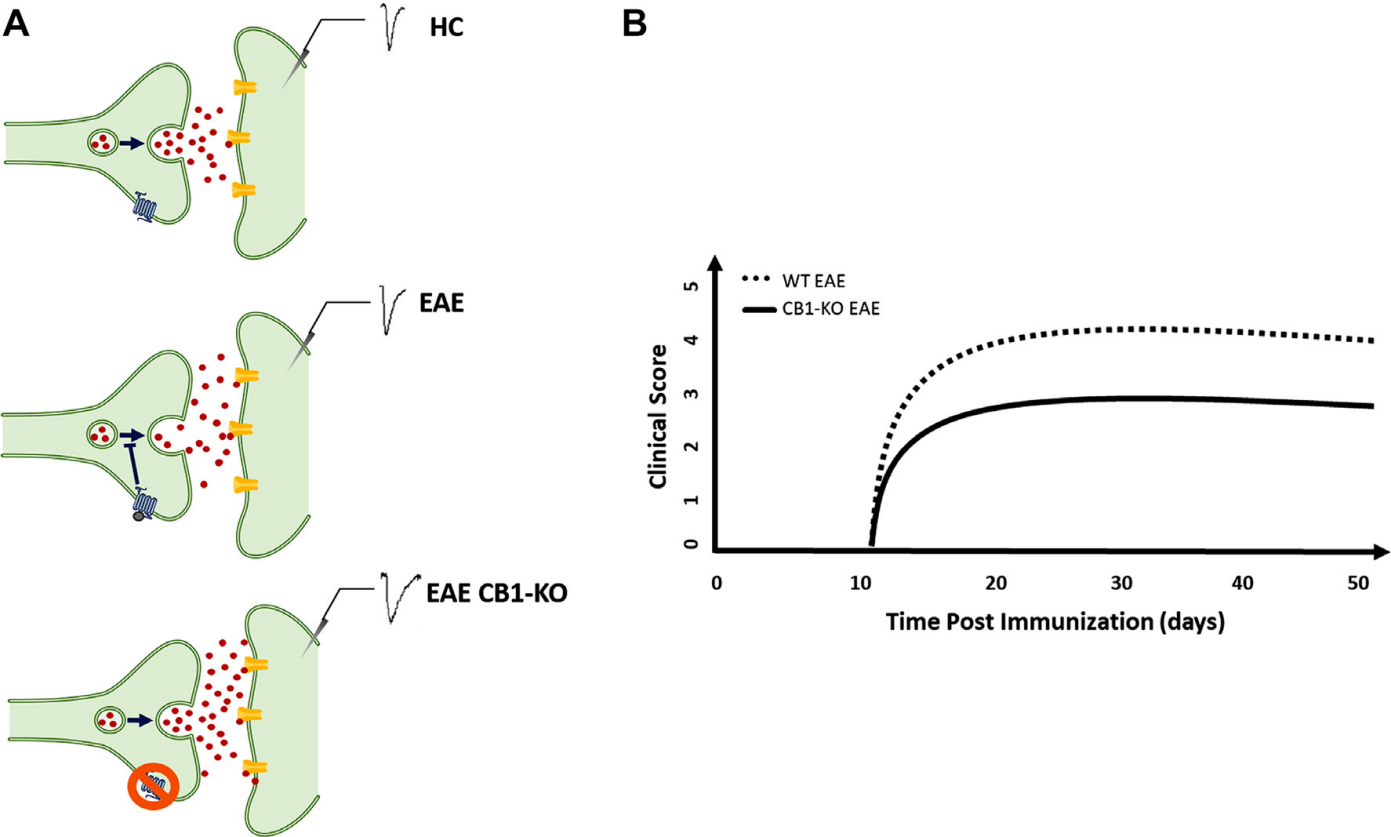
The endocannabinoid system influences synaptic transmission and clinical score in EAE. (**A**) Endocannabinoids released from post-synaptic neurons modulate the release of glutamate in pre-synaptic neurons. An increase in EPSP duration is observed in EAE compared with HC mice as a response to neuroinflammation. EAE CB1R-KO mice exhibit an additional synaptotoxic effect that is observed as a further increase in EPSP duration. (**B**) CB1R-KO EAE mice have a worse clinical score than mice regularly expressing CB1R (original graphic from Rossi *et al*., 2011). **Abbreviations**: experimental autoimmune encephalomyelitis (EAE), excitatory postsynaptic potential (EPSP), healthy control (HC), cannabinoid receptor 1 (CB1R).

**Fig. (2):**
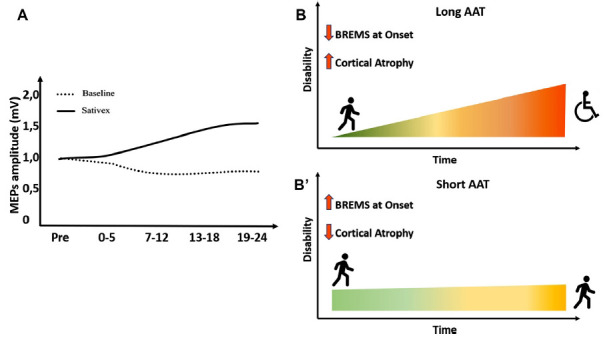
The endocannabinoid system influences synaptic plasticity, brain atrophy, and risk of progression in MS. (**A**) pwMS treated with a cannabis preparation (Sativex^®^) have persistent MEP amplitude enhancement after cTBS treatment, suggesting a neurophysiological effect of cannabinoids in synaptic plasticity phenomena (original graphic from Koch *et al*., 2009). (**B**) PwMS presenting ≥ 12 AAT short tandem repeats (long AAT) in both alleles of the CB1R gene have a higher risk of disease progression at diagnosis, as measured by BREMS, and increased cortical atrophy at the disease onset. (**B’**) PwMS presenting < 12 AAT short tandem repeats (short AAT) in both alleles of the CB1R gene have a lower risk of disease progression at diagnosis, as measured by BREMS, and reduced cortical atrophy at the disease onset. **Abbreviations**: patients with multiple sclerosis (pwMS), motor-evoked potentials (MEP), continuous theta burst stimulation (cTBS), Bayesian Risk Estimate for MS (BREMS).

Long-term potentiation (LTP) is considered the fundamental neurophysiological synaptic mechanism underlying neurological recovery following brain damage [25, 35]. Both acute and chronic brain damage disrupts brain connectivity, leading to the manifestation of neurological signs and/or symptoms of MS [25, 35]. LTP occurs in surviving neurons as a compensatory mechanism for network disconnection that profoundly affects disability [36] and the clinical course of MS [37]. ECS are homeostatic modulators of network activity promoting synaptic plasticity in both EAE and MS [38]. Genetic deletion of CB1Rs in mice results in a worse EAE course and profound synaptic defects [23] (Fig. **1**, **1A**). The administration of nabiximols directly influences synaptic potentiation over depression in pwMS, which was explored through transcranial magnetic stimulation (TMS) [39] (Fig. **2A**). Cannabinoids are bidirectionally implicated in neurological recovery mediated by exercise and motor rehabilitation [40]. CB1R activation indirectly promotes the synaptic plasticity induced by physical exercise in both rodents and humans [35], and exercise enhances endocannabinoids levels in humans, suggesting that the LTP-promoting effects of motor rehabilitation can be partly mediated by the upregulation of the ECS [40]. In a neurophysiological study, it was demonstrated that genetically determined reduction of CB1R expression, caused by the presence of ≥ 12 AAT short tandem repeats (long AAT repeats) in both alleles of the CB1R gene, impairs theta burst stimulation (TBS)-induced cortical plasticity [41] poring clinical response to rehabilitation in MS [35] (Figs. **2B**, **B**). Moreover, pwMS with lower CB1R expression had a higher risk of disease progression, as measured by the functional composite score progression or Bayesian Risk Estimate for MS (BREMS) [42], and greater cortical and optic nerve atrophy at the disease onset [26]. Synaptic plasticity contributes to shaping brain connectivity, and altered synaptic functioning entails pathological modulation of brain networks in MS [43]. Magnetic resonance imaging (MRI) studies of cortical connectivity have recently suggested a possible impact of cannabinoid treatment in the modulation of connectivity between motor areas [44]. On the other hand, a functional MRI (fMRI) study demonstrated that nabiximols do not influence cortical excitability within motor areas in spastic PMS patients [45]. These data suggest that nabiximols affect both nonmotor [45] and motor areas [44], further corroborating its role in modulating other symptoms of SPS. A large body of evidence showed that neuroinflammation directly interacts with synaptic transmission mechanisms, inducing the so-called inflammatory synaptopathy, a hallmark of MS pathophysiology [24, 37, 46]. Interestingly, the interaction between cannabinoids and the inflammatory molecules responsible for synaptic dysfunction has been reported in recent years. For example, the presence of IFN-γ in the striatum of EAE mice reduced the levels of CB1R, contributing to depressive and anxiety behavior [47], and endocannabinoids dampen the effect of both TNF on postsynaptic glutamate receptor expression and function [42] (Fig. **1B**) and IL-1β on presynaptic glutamate release [23]. Moreover, in physiological conditions, the reduction of glutamatergic tone mediated by CB1 in the hippocampus and cerebellum limits the excitotoxic damage mediated by inflammatory molecules and increases the concentrations of brain-derived neurotrophic factor (BDNF) [48]. These effects contribute to the neuroprotective shield against excessive neuronal activation [48].

## 5 DISCUSSION

In the present article, we summarized the existing evidence on the involvement of cannabinoids and nabiximols in a wide range of pathophysiological key mechanisms of MS, including neuroinflammation, synaptic plasticity, and cortical connectivity. A seminal study by Moreno-Martet and colleagues proposed a potential function for nabiximols as a disease-modifying therapy. The authors showed that administering nabiximols-like drugs intraperitoneally at the onset of symptoms and continuing until the first relapse of the disease could mitigate the progression of neurological deficits in EAE [33]. In this respect, nabiximols mechanism of action is not only mediated by an interaction with CB1 and CB2 receptors in neurons of the frontal and prefrontal motor cortex but also involves interference with MS-specific demyelination and axonal pathology [6, 9]. A wide range of DMTs share the ability to influence synaptic transmission and plasticity in MS and EAE, modulating the neuroinflammatory mechanisms involved in synaptic control [37, 49-53]. For example, fingolimod improves functional connectivity in RRMS patients [49], preventing and reversing pre- and postsynaptic alterations of glutamate transmission in EAE mice and reducing neuronal dendritic pathology [50]. Similarly, fingolimod reverted LTP loss, favoring memory and reducing the volume of ischemic lesions in a mouse model of focal cerebral ischemia [54]. Also, cladribine selectively enhanced glutamatergic synaptic transmission and modulated the synaptotoxic effects of IL-1β [51]. Finally, treatment with IFNβ-1a improved cortical plasticity in RRMS patients, resulting in ameliorated cognitive performance [52]. Based on the above results, we suggest that cannabinoids and nabiximols should be considered not just as symptomatic agents against spasticity, but rather as a prototypical DMST. Further studies and clinical trials are needed to explore the role of nabiximols as an add-on therapy to other DMTs and to rehabilitation [55]. Considering that both progression independent from relapse activity (PIRA) and SPS symptoms develop during the early stages of the disease, early treatment with drugs interacting with the cannabinoid system could represent a breakthrough in the treatment of MS symptoms. Unfortunately, treatment with nabiximols is typically prescribed in advanced MS patients when the neurodegenerative process associated with chronic inflammation is already established and irreversible. The good tolerability of this cannabinoid preparation and the better understanding of its DMT potential should favor an early place in the therapy of nabiximols, ideally when the first sign or symptom of the SPS appears. From a future perspective, inhibitors of fatty acid amide hydrolase (FAAH) or of monoacylglycerol lipase (MAGL), which degrade AEA and 2-AG, respectively, are gaining particular interest. MAGL inhibitors were demonstrated to increase the tone of endocannabinoids in EAE mice, slowing EAE disability progression [24]. Similarly, FAAH inhibitors reduced spasticity in a mouse model of both relapsing-remitting and secondary progressive MS [24].

## CONCLUSION

The present article emphasized the key role of the ECS in the modulation of the inflammatory and neurodegenerative processes of MS. Combined with previous evidence demonstrating the efficacy of ECS modulators in the treatment of SPS, suggesting their further role as DMSTs.

## AUTHORS’ CONTRIBUTIONS

A.B., P.A., M.C., E.C., A.C., G.A.M., M.S., V.T., V.T.C., R.T., A.B., D.C: conceptualization; A.B. and D.C., writing; P.A., M.C., E.C., A.C., G.A.M., M.S., V.T., V.T.C., R.T., E.D.: revision.

## LIST OF ABBREVIATIONS

2-AG: 2-Arachidonoylglycerol
BDNF: Brain-derived Neurotrophic Factor
BREMS: Bayesian Risk Estimate for MS
CB1R: Cannabinoid-receptor-1
CBD: Cannabidiol
DMST: Disease-modifying Symptomatic Treatment
DMTs: Disease-modifying Treatments
EAE: Experimental Autoimmune Encephalomyelitis
ECS: Endocannabinoid System
EDSS: Expanded Disability Status Scale
FAAH: Fatty Acid Amide Hydrolase
fMRI: Functional MRI
IFN: Interferon
IL: Interleukin
LTP: Long-term Potentiation
MAGL: Monoacylglycerol Lipase
MRI: Magnetic Resonance Imaging
MS: Multiple Sclerosis
pwMS: Patients with MS
PIRA: Progression Independent from Relapse Activity
PMS: Progressive MS
RRMS: Relapsing-remitting Multiple Sclerosis
SPS: Spasticity Plus Syndrome
ST: Symptomatic Therapies
TBS: Theta Burst Stimulation
THC: Delta-9-tetrahydrocannabinol
TMS: Transcranial Magnetic Stimulation
